# Association of Ramadan Fasting and Clinical Outcomes in Patients With Myasthenia Gravis

**DOI:** 10.1001/jamanetworkopen.2020.4373

**Published:** 2020-04-23

**Authors:** Ismail Ibrahim Ismail, Fathi Massoud Abokalawa, Walaa Kamel, R. Khan, Jasem Youssef Al-Hashel

**Affiliations:** 1 Department of Neurology, Ibn Sina Hospital, Sabah Medical Area, Kuwait City, Kuwait; 2 Department of Neurology, Beni-Suef University, Beni-Suef, Egypt; 3 Health Sciences Centre, Department of Medicine, Faculty of Medicine, Kuwait University, Kuwait City, Kuwait

## Abstract

This cohort study examines the association of Ramadan fasting with clinical outcomes in patients with myasthenia gravis.

## Introduction

Ramadan fasting (RF) is when millions of Muslims abstain from food and drink from dawn until sunset.^1^ Patients with chronic conditions who are eligible for exemption sometimes insist on fasting without seeking medical advice.^2^ To our knowledge, the effects of RF on myasthenia gravis (MG) have never been studied, and neurologists usually refrain from advising patients with MG to fast because of the lack of evidence-based knowledge regarding its safety. We aimed to study the association of RF with clinical outcomes in patients with MG and to use clinical characteristics to estimate outcome risk.

## Methods

This prospective cohort study was conducted in the main tertiary neurology center at Ibn Sina Hospital in Kuwait from April through June 2019 (Ramadan began May 5 and ended June 3). The study was approved by the institutional review board of the Ministry of Health of the state of Kuwait. All patients provided written informed consent to participate in the study and for their anonymized data to be used for analysis. This study follows the Strengthening the Reporting of Observational Studies in Epidemiology (STROBE) reporting guideline.

Patients with MG who were willing to fast were evaluated through 3 clinical visits: 1 month before Ramadan, during the last week of Ramadan, and 1 month after Ramadan. Disease severity was classified according to Myasthenia Gravis Foundation of America clinical classification from class I to class V.

Outcomes were classified as stable, worsened, or improved disease according to changes in patients’ Myasthenia Gravis Foundation of America clinical class during RF. Patients were instructed to break their fasting in case of worsening.

Data were analyzed using SPSS statistical software version 24.0 (IBM). Significance was determined with 2-sided χ^2 ^tests or the Mann-Whitney*U* test, with the significance level set at *P* < .05. A receiver operating characteristic curve was used to determine a cutoff point to estimate outcome. Data were analyzed from September 2019 to October 2019.

## Results

We evaluated 141 patients with MG (mean [SD] age, 46.8 [15.1] years; 58 men [51.3%]) in the month before Ramadan. Of the patients, 113 (80.1%) completed the study; 93 (82.3%) had generalized MG, and the mean (SD) time since disease onset was 7.4 (6.7) years. Fasting duration was 15 hours per day for 29 days. The outcomes were stable disease in 91 patients (80.5%) and worsened disease in 17 patients (15.0%) (Table), including 2 patients who developed severe weakness of limb and axial muscles; 5 patients (4.4%) had improved disease. Of the patients whose conditions worsened during Ramadan, 15 (94.1%) returned to pre-Ramadan status during the follow-up period.

**Table.** Comparison of Patients With Myasthenia Gravis Whose Conditions Remained Stable or Worsened During Ramadan Fasting

Clinical parameter	Patients, No. (%)		Test of significance	*P* value
	Stable (n = 91)	Worsened (n = 17)		
Age, y				
Median (range)	48.0 (14.0-79.0)	45.0 (22.0-75.0)	*U* = 760.5	.91
Mean (SD)	46.2 (15.3)	47.2 (15.2)		
Sex				
Male	44 (48.4)	11 (64.7)	χ^2^ = 1.533	.21
Female	47 (51.6)	6 (35.3)		
Time since onset, y				
Median (range)	5.0 (1.0-35.0)	3.0 (1.0-29.0)	*U* = 580.0	.10
Mean (SD)	7.4 (6.5)	6.4 (7.1)		
Type				
Generalized	72 (79.1)	16 (94.1)	χ^2^ = 4.307^a^	.03^a^
Ocular	18 (19.8)	1 (5.9)		
Acetylcholine receptor antibodies				
Positive	65 (71.4)	15 (88.2)	χ^2^ = 2.107	.22
Negative	26 (28.6)	2 (11.8)		
Thymic hyperplasia	55 (60.4)	10 (58.8)	χ^2^ = 0.016	.90
Thymoma	1 (1.1)	1 (5.9)	χ^2^ = 1.803	.29
Thymectomy	43 (47.3)	9 (52.9)	χ^2^ = 0.186	.79
Pre-Ramadan disease severity				
Normal	66 (72.5)	7 (41.1)	χ^2^ = 21.719^a^	<.001^a^
Myasthenia Gravis Foundation of America class				
I	14 (15.4)	2 (11.8)		
IIa	7 (7.7)	2 (11.8)		
IIb	4 (4.4)	1 (5.9)		
IIIa	0	3 (17.6)		
IIIb	0	2 (11.8)		
Pyridostigmine dose, mg/d				
Median (range)	180.0 (60.0-450.0)	270.0 (160.0-600.0)	*U* = 256.0^a^	<.001^a^
Mean (SD)	190.4 (70.2)	298.2 (106.3)		
Pyridostigmine frequency before Ramadan, doses/d, No.				
Median (range)	3.0 (1.0-5.0)	4.0 (3.0-5.0)	*U* = 282.50^a^	<.001^a^
Mean (SD)	3.3 (0.8)	4.3 (0.7)		
Other immunosuppressants	66 (72.5)	16 (94.1)	χ^2^ = 3.653	.06
Azathioprine	40 (44.0)	11 (64.7)	χ^2^ = 2.475	.11
Prednisolone	38 (41.8)	3 (17.6)	χ^2^ = 3.536	.04^a^
Mycophenolate mofetil	12 (13.2)	2 (11.8)	χ^2^ = 0.026	>.99
Intravenous immunoglobulin	4 (4.4)	1 (5.9)	χ^2^ = 0.072	.58
Rituximab	2 (2.2)	0	χ^2^ = 0.381	>.99
No. of immunosuppressants				
0	25 (27.5)	1 (5.9)	χ^2^ = 14.437^a^	<.001^a^
1	53 (58.2)	6 (35.3)		
2	13 (14.3)	10 (58.8)		
Comorbid diseases	30 (33.0)	6 (35.3)	χ^2^ = 0.035	.85
Diabetes	16 (17.6)	4 (23.5)	χ^2^ = 0.336	.51
Thyroid disease	4 (4.4)	2 (11.8)	χ^2^ = 1.482	.23
Hypertension	13 (14.3)	1 (5.9)	χ^2^ = 0.897	.69
Renal disease	1 (1.1)	0	χ^2^ = 0.189	>.99
Migraine	7 (7.7)	2 (11.8)	χ^2^ = 0.311	.63
Systemic lupus erythematosus	2 (2.2)	0	χ^2^ = 0.381	>.99

Abbreviation: *U*, Mann-Whitney *U* test.

^a^ Statistically significant at *P* ≤ .05.

There was no statistically significant difference between patients with stable vs worsened disease regarding age (mean [SD], 46.2 [15.3] years vs 47.2 [15.2] years), sex (44 men [48.4%] vs 11 men [64.7%]), time since disease onset (median [range], 5.0 [1.0-35.0] years vs 3.0 [1.0-29.0] years), acetylcholine receptor antibody status (65 patients [71.4%] vs 15 patients [88.2%] positive), thymic abnormalities (55 patients [60.4%] vs 10 patients [58.8%] with thymic hyperplasia; 1 patient [1.1%] vs 1 patient [5.9%] with thymoma), thymectomy (43 patients [47.3%] vs 9 patients [52.9%]), or the presence of comorbid diseases (30 patients [33.0%] vs 6 patients [35.3%]). However, we found statistically significant differences between patient groups according to MG type (72 patients [79.1%] vs 16 patients [94.1%] with generalized MG; 18 patients [19.8%] vs 1 patient [5.9%] with ocular MG; χ^2^ = 4.307; *P* = .03), disease severity before Ramadan (66 patients [72.5%] vs 7 patients [41.1%] with normal disease severity; χ^2^ = 21.719; *P* < .001), pyridostigmine dose (median [range], 180.0 [60.0-450.0] mg per day vs 270.0 [160.0-600.0] mg per day; *U* = 256.0; *P* < .001), pyridostigmine frequency (median [range], 3.0 [1.0-5.0] doses per day vs 4.0 [3.0-5.0] doses per day; *U* = 282.50; *P* < .001), number of immunosuppressants (13 patients [14.3%] vs 10 patients [58.8%] taking 2 immunosuppressants; χ^2^ = 14.437; *P* < .001), and taking prednisolone (38 patients [41.8%] vs 3 patients [17.6%]; χ^2^ = 3.536; *P* = .04) (Table). Patients taking pyridostigmine with a dose of 240 mg or less daily (area under the receiver operating characteristic curve, 0.792; 95% CI, 0.698-0.886; *P* < .001), patients taking pyridostigmine 3 times or less daily (area under the receiver operating characteristic curve, 0.779; 95% CI, 0.683-0.875; *P* < .001), and patients whose disease was Myasthenia Gravis Foundation of America severity class IIb or lower before Ramadan (specificity, 100%; positive predictive value, 100%; χ^2^ = 28.064; *P* < .001) were statistically significantly more likely than patients without those characteristics to have a stable outcome during fasting.

## Discussion

To our knowledge, this study represents the first description of clinical outcomes of patients with MG during RF in the literature. Ramadan fasting appears to be safe and well tolerated for most patients with MG in our cohort. Only 15.0% showed worsening of their symptoms; however, no patient developed respiratory involvement or myasthenic crisis and only 2 patients developed severe weakness of limb and axial muscles. This is in line with previous studies^3^ showing that 10% to 15% of patients with MG will have worsening disease status despite conventional treatment regimens.

A favorable outcome was observed in patients with ocular MG, those with generalized MG of class IIb or lower before Ramadan, those taking pyridostigmine with a dose of 240 mg or less per day at a frequency of 3 times or less per day, those taking prednisolone, and those who needed only 1 immunosuppressant to control their symptoms. We propose a risk assessment scheme using these clinical parameters to classify patients with MG as having low, moderate, and high risk for fasting with possible treatment adjustments (Figure).

**Figure.**
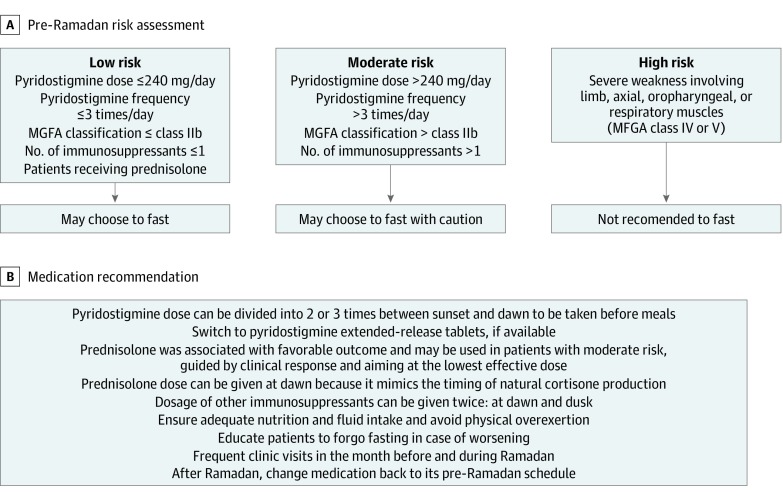
Proposed Scheme for Risk Stratification of Patients With Myasthenia Gravis for Ramadan Fasting Charts show pre-Ramadan risk assessment (A) and medication recommendations (B). MGFA indicates Myasthenia Gravis Foundation of America.

Moreover, most patients whose conditions worsened during Ramadan returned to pre-Ramadan status during the follow-up period. This finding is in line with data from systematic reviews showing that RF only mildly influences the immune system and that the changes are transient and return to pre-Ramadan status shortly afterward.^4,5^

The ability of patients with MG to observe a total or a partial fasting during Ramadan can be attributed to the diurnal variation of weakness, which is a characteristic feature of MG. Other possible reasons might be the decrease in physical exertion, reversed sleep pattern, and shortened working hours during Ramadan.

A limitation of this study is that patients were not able to use pyridostigmine extended-release tablets because of their unavailability in Islamic countries. Moreover, the generalizability of our results needs further multicenter studies with larger cohorts to confirm our results.
