# Error in Author’s Surname

**DOI:** 10.1001/jamanetworkopen.2020.10013

**Published:** 2020-05-29

In the Research Letter titled “Association of Ramadan Fasting and Clinical Outcomes in Patients With Myasthenia Gravis,”^1^ published April 23, 2020, the surname of the third author was misspelled as Kamel. The correct spelling is A.Kamel. This article has been corrected.^1^
